# Metamaterial Superlenses Operating at Visible Wavelength for Imaging Applications

**DOI:** 10.1038/s41598-018-33572-y

**Published:** 2018-10-31

**Authors:** S. Haxha, F. AbdelMalek, F. Ouerghi, M. D. B. Charlton, A. Aggoun, X. Fang

**Affiliations:** 1 Royal Holloway, University of London, Microwave Photonics and Sensors group Department of Electronic Engineering, Egham, Surrey, TW20 0EX United Kingdom; 2 Advanced Materials and Quantum Phenomena Laboratory, Faculty of Science at Tunis, University of El Manar, Tunis, Tunisia; 3 Electronics and Computer Science, University of Southampton University Road Southampton, SO17 1BJ Southampton, United Kingdom; 4 School of Mathematics and Computer Science, University of Wolverhampton Wulfruna Street Wolverhampton, WV1 1LY Wolverhampton, United Kingdom

## Abstract

In this paper, a novel design for a metamaterial lens (superlens) based on a Photonic Crystal (PC) operating at visible wavelengths is reported. The proposed superlens consist of a gallium phosphide (GaP) dielectric slab waveguide with a hexagonal array of silver rods embedded within the GaP dielectric. In-house 2DFDTD numerical method is used to design and optimize the proposed superlens. Several superlenses are designed and integrated within a same dielectric platform, promoting the proof-of-concept (POC) of possible construction of an array of superlenses (or sub-lenses to create an M-Lens) for light field imaging applications. It is shown that the concavity of the superlens and positioning of each sub-lens within the array strongly affects the performances of the image in terms of resolution. Defects and various geometrical shapes are introduced to construct and optimize the proposed superlenses and increase the quality of the image resolution. It is shown that the orientation of the active region (ellipse) along *x* and *y* axis has tremendous influence on the quality of image resolution. In order to investigate the performance characteristics of the superlenses, transmitted power is calculated using 2D FDTD for image projections at various distances (in *x* and *y* plane). It is also shown, how the proposed superlens structures could be fabricated using standard micro fabrication techniques such as electron beam lithography, inductively coupled Reactive ion etching, and glancing angle evaporation methods. To the best of our knowledge, these are the first reported POC of superlenses, integrated in a monolithic platform suitable for high imaging resolution that can be used for light field imaging applications at visible wavelength. The proposed superlenses (integrated in a single platform M-Lens) will have tremendous impact on imaging applications.

## Introduction

Emerging superlenses with aberration correction have generated great attention in the field of the optical microscopy and imaging applications due to their ability to significantly increase the image resolution quality. These superlenses provide super image resolution by overcoming the classical diffraction limits of light propagation^1–3^. Although many lens imaging devices and systems have been realized, the negative refraction mechanism offered by Photonic Crystals (PC) devices enable high quality resolution at nanoscale^4^. Surface plasmon-polaritons based on PC structures offer a unique imaging lens approaches such as hyperlenses^5–9^ and/or metamaterial superlenses^10,11^. Advantages in understanding of the fundamental mechanisms of the light propagation has enabled a rapid progress in developing of devices and systems that offer high quality image resolution. These advanced light propagation control and manipulation approaches have provided unprecedented high quality resolution images.

PC structures, and metamaterial lenses based on PC structures for light coupling, light manipulations, photonic integration, biosensing and various imaging applications have been extensively reported in the literature^12–20^. Modulating dispersion properties of PC based lens with low index and tunable optical properties using various microfluidics and as used as an optical coupling element operating a various wavelengths (0.75–3 μm) have been discussed in^12^. Spatially-variant PC structures for integrated photonics, where the alignment of the unit cell varies as a function of position capable of sharply guiding light beams using just low index materials with very high polarization selectivity is reported in^13^. In this ref.^13^, a PC structure configuration for controlling the light is proposed where the infrared light of one polarization is propagating through sharp bends, whereas the other polarization of the light is propagating straight through the spatial variant PC. PC lens based on ultra-sharp nano-focusing of graded index with optimized single defect and with the beam spot size (Full width at half maximum (FWHM) = λ/75) that could be used for fluorescent imaging analysis, biosensing and sub-surface nano-metrology applications are reported in^14^. A PC lens structure with high coupling efficiency is reported in ref.^15^, despite differences between the theoretical and experimental obtained results, the reported PC lens^15^ can still be used as an effective method for light coupling in THz integrated circuits. A PC planar lens operating at microwave (low) frequencies using the ray tracing method has been reported in^16^. Various designs, using FEM-based numerical simulations, of plano-concave lenses with epsilon-near-zero surface-relief coatings for efficient shaping of nonparaxial optical beams are reported in^17^. Plano-concave silicon lenses based on coupled metacoatings set at the entrance and exit surfaces of a transparent dielectric thick lens structure with a potential applications in optical trapping and detection is reported in^18^. In^19^ two types of binary 2D subwavelength (operating at wavelength was λ = 10 mm) focus diffractive PC lens is reported. Numerous PC types of structural disorders in the hole diameter and to interface roughness, including the imperfection disorder in the lattice periodicity in terahertz time-domain spectroscopy are reported in^20^. It is reported that guided resonances in the PC lens structures are prone to any potential hole diameter disorders and interface roughness, nevertheless they are more sensitive to lattice periodicity fabrication imperfections. It should also be noted that, in our previous work reported in^21^, we have reported a planoconcave metamaterial lens based on PC structure for integration with a Single Mode Fiber (SMF) and an ordinary photonic crystal waveguide.

In this study, we have proposed a novel lens with unique structure based on a combination of metamaterials and engineering of PC structure geometry shape, defects^11^, and introduction of reflectors are included. By altering and optimising various lens structure parameters, the ray focus position at any desired location would have significant contributions to light field imaging applications. Light field imaging technology was introduced by Lippman in 1908^22^. Light field imaging emulates the visual system of insects, specifically the fly, where micro-lenses are used to capture an object section or a part, enabling 3D content imaging by using a single camera snapshot. This technique offers improved imaging while enhancing the 3D view without any additional visual assistance. Light field has been attracting a significant amount of interest in recent year due to its property to provide refocus after shoot, depth computation and 3D replay^23–25^. An important observation from the Lipman concept is that the replayed image captured through an micro-lens array will result in an inverted depth^26^. Previous solutions use a double micro-lens array to invert the image before recording which makes the capture system cumbersome^26^. Metamaterials are new types of materials which refract light in the opposite direction to conventional materials. Hence, a negative refractive index metamaterial refracts the light on the same side of the normal as the incident beam, rather than on the opposite side as in conventional materials. Using a micro-lens array where each micro-lens is made of negative refractive index metamaterial lens will resolve this problem. Example of Raytrix Light field imaging system uses micro-lens arrays with various micro lens forms which vary in their focal length to allow extension of the depth of field. Using conventional micro-lens structure will make the fabrication process complex. Using Metamaterials allows several similar/identical or different superlens platforms can be designed depending on the applications and hence offers greater opportunities to extend the depth of field for greater depth computation.

Using Metamaterials allows several similar/identical or different superlens platforms can be designed depending on the applications and hence offers greater opportunities to extend the depth of field for greater depth computation. Current conventional microlens arrays suffer from high losses, low (poor) resolution, inability to determine the depth of an object in the third dimension, and have limited angle of view (AOV)^27–31^. It is also known that the resolution of current optical devices is limited by the wavelength of light. In particular, conventional lenses are unable to image objects smaller than half the illumination wavelength. The proposed superlens would have significant advantages when capturing an object image, and can be designed to fit easily within a conventional pixel size. Therefore our proposed metamaterial superlenses will have great impact on enhancing the resolution and projection of light field imaging system. The proposed lens based on metamaterial PC structure would provide significant enhanced image processing functionality. By engineering the geometry and optimizing the design parameters of the PC structures, the focus of the light can be tuned to the surface of the lens where the novel configuration of metasurface is able to overcome the light diffraction limits. Along the propagation of the light, the wavefront of the waves is constructed where the light can be used to generate a high imaging resolution. Since, the light propagates along the PC structure, its intensity decays, when it interacts with a sub-wavelength aperture, the intensity increases and the collection of the light wavelength can be performed. The proposed superlens will enable and simplify image transformations such as rotation, orientation, scaling and lighting changes. To the best of our knowledge, this is the first reported superlenses that can be integrated in an array suitable for high imaging resolution operating at visible wavelength. Designing and integrating an array of precise superlenses into a single lens platform will lead to a new class of imaging lens systems which will exhibit high resolution beyond existing state-of-the-art^27^.

## Simulation Method

The proposed PC based superlens structures are simulated by using in-house 2D Finite Difference Time Domain (FDTD) method^32^. In order to design, analyze and optimize the proposed PC superlens structure parameters, we have employed our in-house accurate 2D FDTD with a computational window enclosed by Perfectly Matched Layers (PML)^33–37^. The FDTD method^33^ is the most effective established numerical method for designing of metamaterial PC based devices. The FDTD method is considered a simple numerical method that enables modelling of complex periodic structures by solving directly using Maxwell’s equation. The foundation of this method was proposed by Yee^34^. In ref.^34^ the author used differential and integral Maxwell’s equations to represent the geometrical spatial sampling of the vector components of the magnetic and electric fields. The numerical technique introduced in ref.^34^ represents the FDTD time and space second order accuracy, where the numerical dispersion effects can be kept at a smaller size than *λ*/20 or *λ*/30. The stability of the FDTD algorithm in ref.^34^ requires an upper bound on the time step (∆*t*) that is determined by the spatial increments ∆*x*, ∆*y*, and ∆*z* in accordance.

## Structure Fabrication Method

The proposed PC superlens structure could be fabricated using standard micro fabrication techniques such as electron beam lithography, inductively coupled Reactive ion etching, and glancing angle evaporation methods. In practice the device would be implemented as a three layer slab waveguide providing a Planar Light Circuit (PLC) platform for device testing. This would consist of a thin (400 nm) GaP core layer grown epitaxially on a transparent Sapphire substrate, which provides a transparent underlying waveguide buffer layer. An overcoat top layer of SiO2 would be deposited by Plasma Enhanced Chemical Vapour Deposition (PECVD) or reactive sputtering, to form a top cladding layer. The pattern consisting of an array of holes would be defined using electron-beam lithography according to the above layout. The pattern would be transferred through the SiO2 to the GaP layer by inductive coupled reactive ion etching using CHF3 and BCl3 dry etch chemistry and a durable resist mask (ZEP). The Inductive coupled plasma process conditions can be adjusted to provide straight vertical side walls through the volume of the core layer (aspect ratios of 4:1 are easily achievable). With the remaining resist mask in place, the structure would then be coated with a thin silver layer by glancing angle e-gun evaporation. This ensures preferential coating of the inside walls of the holes as opposed to the top and bottom surfaces. Thickness is controlled to nm precision by a crystal monitoring system. For very thin conformal layers, electroplating could be used to grow the silver rings to the required thickness. Resist would then be removed by conventional metal lift off process to reveal a clean metal free top SiO2 surface.

Finally the entire structure would be over-coated with GaP material by reactive sputtering process to back-fill the central part of the holes. This will overcoat the top SiO2 surface as well, however this since layer is optically isolated from the GaP waveguide core by the top SiO2 layer, it does not interfere with optical transmission through the underlying GaP core layer. If necessary the residual GaP layer could be removed from the top surface by a planarization etch.

To couple light into the device grating couplers would be etched into the PLC to couple light into the device from the top. Scanning Near field Optical Microscopy (SNOM) would be used to test operation of the device. This involves positioning a tapered optical fiber over the top of the device, which in turn probes the evanescent field or scattered leakage light, and so provides a method to map out the optical field distribution in a practical way, for comparison to the field maps above.

## Dispersion in Ag Array

It is already know that in PC structures that consist of metals, such as silver cylinders considered in this study, most electrons are free since they are not bound to the nucleus, therefore the restoring force is insignificant and there is no natural frequency occurring. Hence for the silver material dispersion, we have derived the Drude model by assuming;

1 $${\varepsilon }_{r}(\omega )={\varepsilon }_{\infty }-\frac{{\omega }_{p}^{2}}{{\omega }^{2}+i\omega {\rm{\Gamma }}}$$

This equation (2) can be written in terms of the real and imaginary components.

2 $${\varepsilon }_{r}(\omega )=1-(\frac{{\omega }_{p}^{2}{\tau }^{2}}{1+{\omega }^{2}{\tau }^{2}})+j(\frac{{\omega }_{p}^{2}\tau /\omega }{1+{\omega }^{2}{\tau }^{2}})$$

where the volume plasma frequency *ω*_*p*_ represents the effective mass of the electron *m* * and its density *N*, hence *ω*_*p*_ = (*Ne*^2^*/ε*_0_ *m* *)1*/*2. The relaxation rate Г defines the effective electron scattering rate, with a matching relaxation time *τ = *1*/*Г, *ε*_∞_ represents the net contribution from the positive ion cores. For the ideal free electron gas *ε*_∞_ = 1, and for metals *ε*_∞_ = 1−10 depending on the interband response^38,39^.

## Results and Discussions

**Figure 1:**
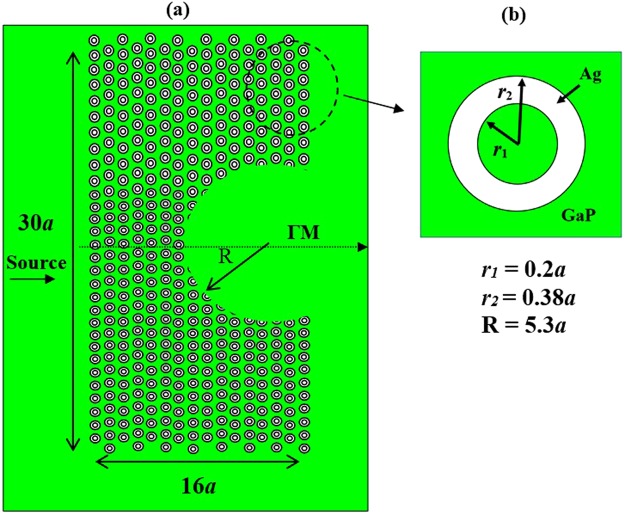
Schematics of the proposed PC metamaterial lens: (**a**) structure geometry consisting of a hexagonal array of GaP/Ag/GaP; (**b**) inset of the unit cell of the periodic structure. The inner radius *r*_1_, outer radius *r*_2_ and the radius *R* of the concave region are defined in the image.

The proposed PC lens structure is shown in Fig. 1, which consists of a hexagonal PC lattice made of silver rods (Ag) embedded in a dielectric background (GaP) material. The dielectric constant, ε_GaP_ of the GaP is 12.25 and the radii of the Ag rods are equal to *r*_1_ = 0.2*a* and *r*_2_ = 0.38*a*, where *a* is the lattice constant and it is equal to 142 nm. The operating wavelength is *λ*_0_ = 483 nm in the blue regime. In the right side of the PC structure, a concave region with radius *R* is introduced as a defect. The dimensions of the structure in *x* and *y* direction are 16 × 142 nm and 30 × 142 nm, respectively, and *R = *5.3*a*. The source is located at 284 nm from the input of the PC metamaterial lens and the thickness of the simulation PML is fixed at 12 layers.

The dynamic process of the proposed PC structures are simulated with the following mesh sizes structure; *dx* = *dy* = *a*/30, where *a* is the lattice constant of the PC structure. In our calculations, we have used the Transverse Magnetic (TM) mode with the ***E***_***z***_, ***H***_***x***_ and ***H***_***y***_ components. The incident light is a broad Gaussian source centered at the frequency *f* = 0.293 *a*/*λ*. At the output of the PC structure a detector is placed to measure the light intensity. The transmission spectra along with the light intensity are determined at this frequency.

In this PC lens structure, the hexagonal PC lattice is selected based on the fact that they exhibit higher symmetry when compared to the PC rectangle lattice. The Brillouin zone is hexagone and its band gap is complete for both polarizations TE and TM. Hexagon PC lattice is larger than rectangle PC lattice. Also, the hexagonal PC lattice will be able to confine the light in 3^rd^ dimension when it is designed in 3D. The selection of the inner and outer radius of Ag rod is based on the focusing of the emerging beam, the optimal dimensions are achieved when the image field is almost similar to the source. The selection of inner and outer radius of the Ag rod and the frequency band enables this structure to behave as metamaterial lens operating at visible wavelength.

**Figure 2:**
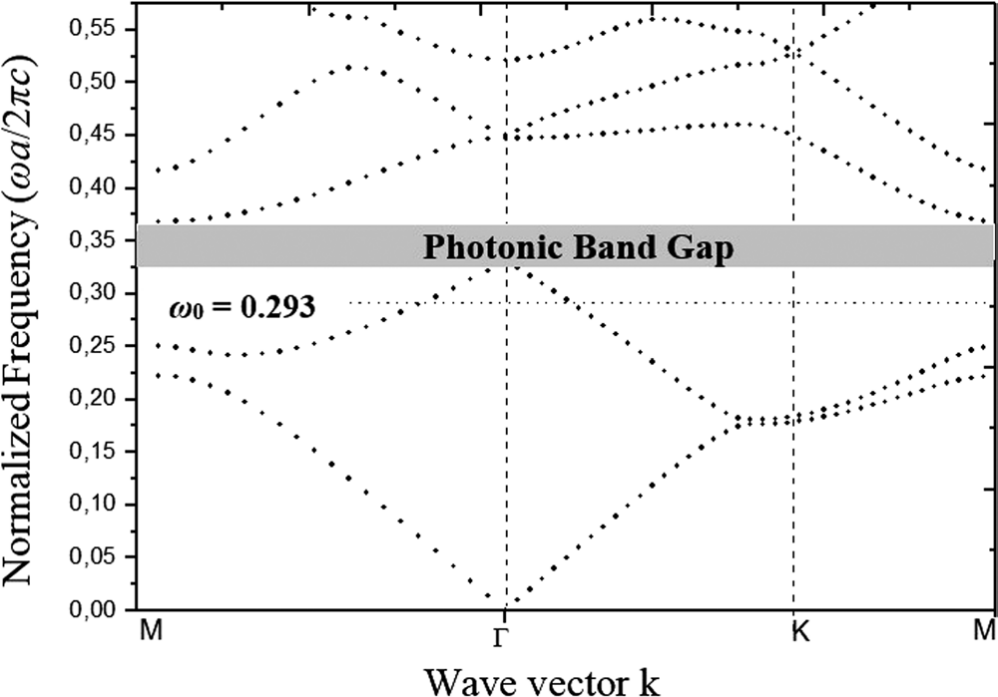
TM band diagram of the proposed metamaterial structure.

The metamaterial lens light behaviour, illustrated in Fig. 2, is fundamentally different from the behaviour of a conventional fully dielectric 2D PC structure. The use of GaP/Ag/GaP materials, strongly modifies the light behaviour for the TM polarisation.

**Figure 3:**
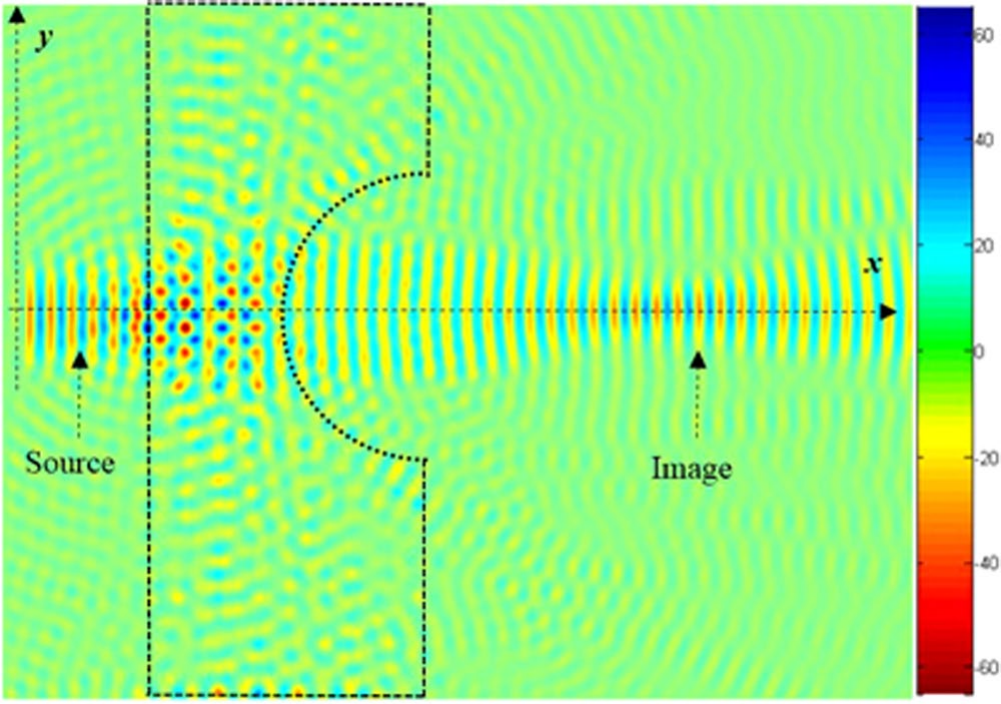
Superlens obtained image at *λ*_0_ = 0, 483 μm, when the point source is 284 nm and a focus is obtained at 3, 6 μm from the source.

By analysing the photonic band diagram of the metamaterial lens, we found that the frequency region of the effective negative refraction exists in the second band in the visible domain with the following parameters; *μ* = 0.293, *λ*_0_ = 0.483 μm, as shown in Fig. 2. In this case, the gradient of the bands indicates that the electromagnetic waves propagate with negative group velocity, and evanescent waves can be supported to perform high image resolution. In other words, significant image resolution is obtained at visible region by carefully designing and optimizing geometrical parameters of the structure shown in Fig. 1. It is worth stating that the quality and size of the formed image is depending on the following condition; $({\overrightarrow{{\rm{V}}}}_{{\rm{g}}}\cdot \overrightarrow{{\rm{k}}}) < 0$ and V_φ_ · V_g_) < 0 where ${\overrightarrow{{\rm{V}}}}_{{\rm{g}}}$ is the group velocity, $\overrightarrow{k}$ is the wave vector and *Vφ* is the phase velocity. When constructing the structure geometry, initially as a first step, we remove silver rods to form *a* in concave defect, and simulate the snap shot of the electric field. The obtained image of the source located on the left of the PC structure at distance *a* is illustrated in Fig. 3.

**Figure 4:**
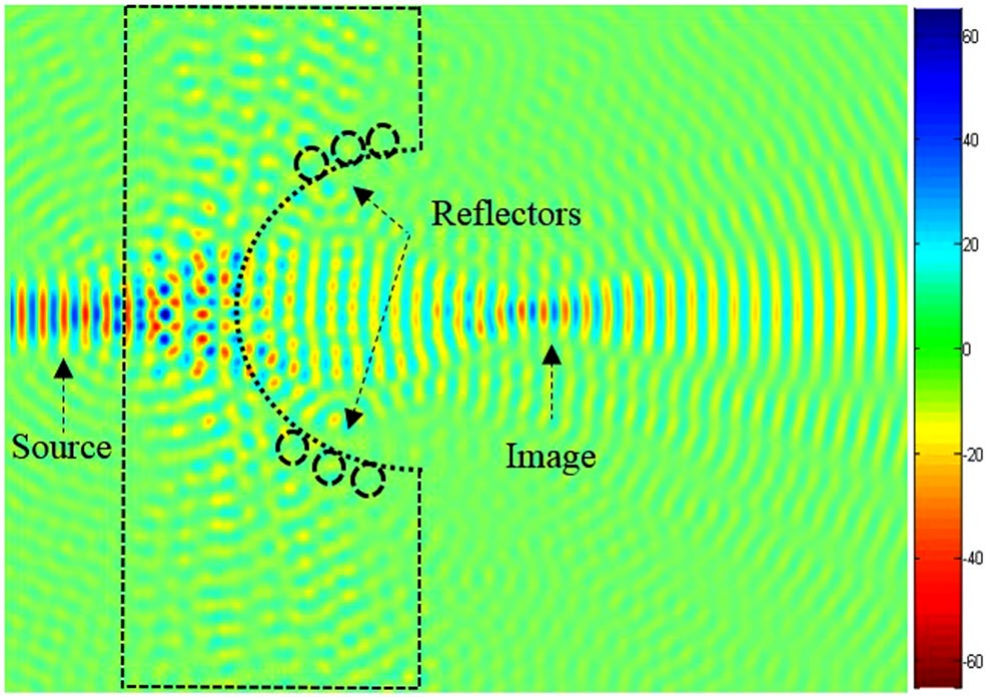
Reconstructed image of the transmitted field at 0, 483 μm, with a radius *R′* = 0.28*a* of the reflector rods. Focusing is observed at 3, 6 μm away from the source. The reflectors shown in the inset picture are based on GaP rods.

In order to increase the resolution of the superlens, we have introduced reflectors on the concave region as illustrated in Fig. 4. The reflectors are rods with larger radius compared to the previous ones, which are considered as defects. While still keeping the source at the same distance *a* from the PC structure, it is clear from this figure that the image is well confined when defect radius is *R*′ = 0.28*a*. Throughout this work *R′* is set to be equal to 0.28*a*.

The promise of perfect aberration free, ultra-high resolution optical imaging could revolutionize nanofabrication technology. To this end, we design a structure enabling perfect optical imaging where the structure is bounded by a reflector composed of GaP rods, as shown in Fig. 4. As can be seen from this figure, the image is clearly formed.

**Figure 5:**
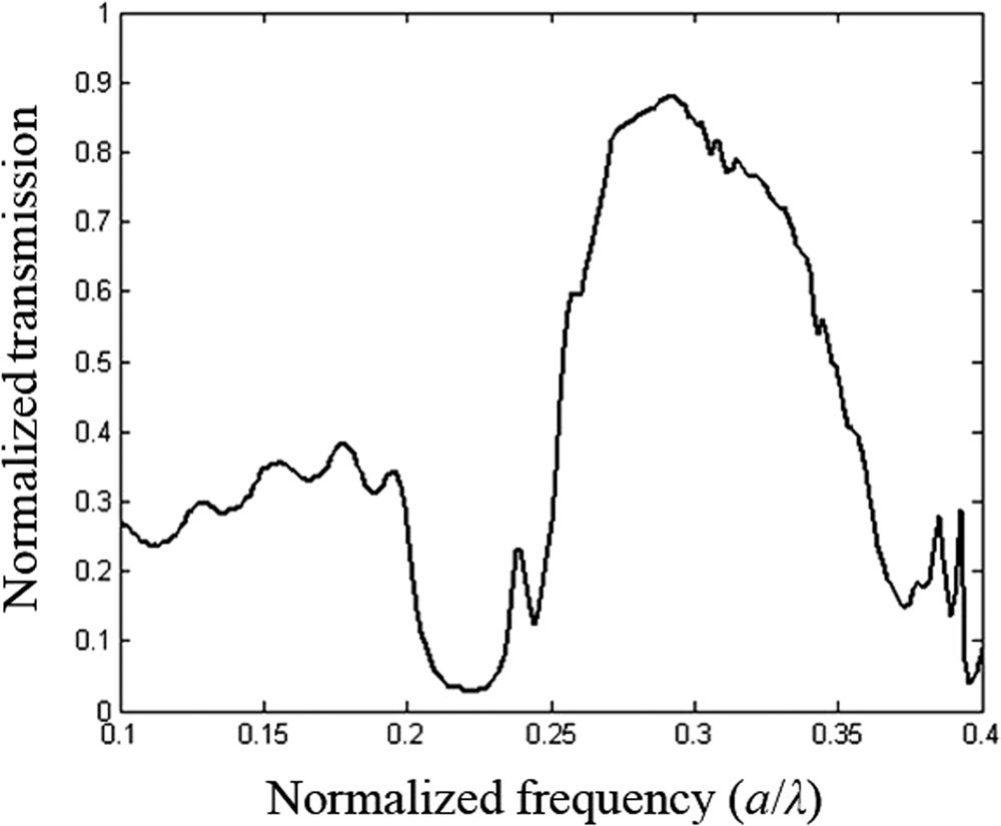
Transmission spectra centred at *λ*_0_ = 0.483 μm for the TM polarization, including the upper and lower reflectors with a radius *R*′ = 0.28*a*. The propagation direction is ΓK.

Figure 5 shows the transmission spectra of the light propagating through the PC lens. As can be seen in this spectrum, the amplitude of the transmission reaches 90% centered at 0.29*a/λ* when the incident light is polarized parallel to rods (TM polarization). While in the spectral range 0.2 *a/λ* and 0.25 *a/λ*, the amplitude is significantly decreased and drops to zero. The diffraction losses of quasi-guided modes are found to be complex functions of mode index and the wavevector. Losses depend on triangular lattice filling fraction, this strongly affects the quality of the image resolution. However, these diffraction losses have impact on the imaging process^40^.

**Figure 6:**
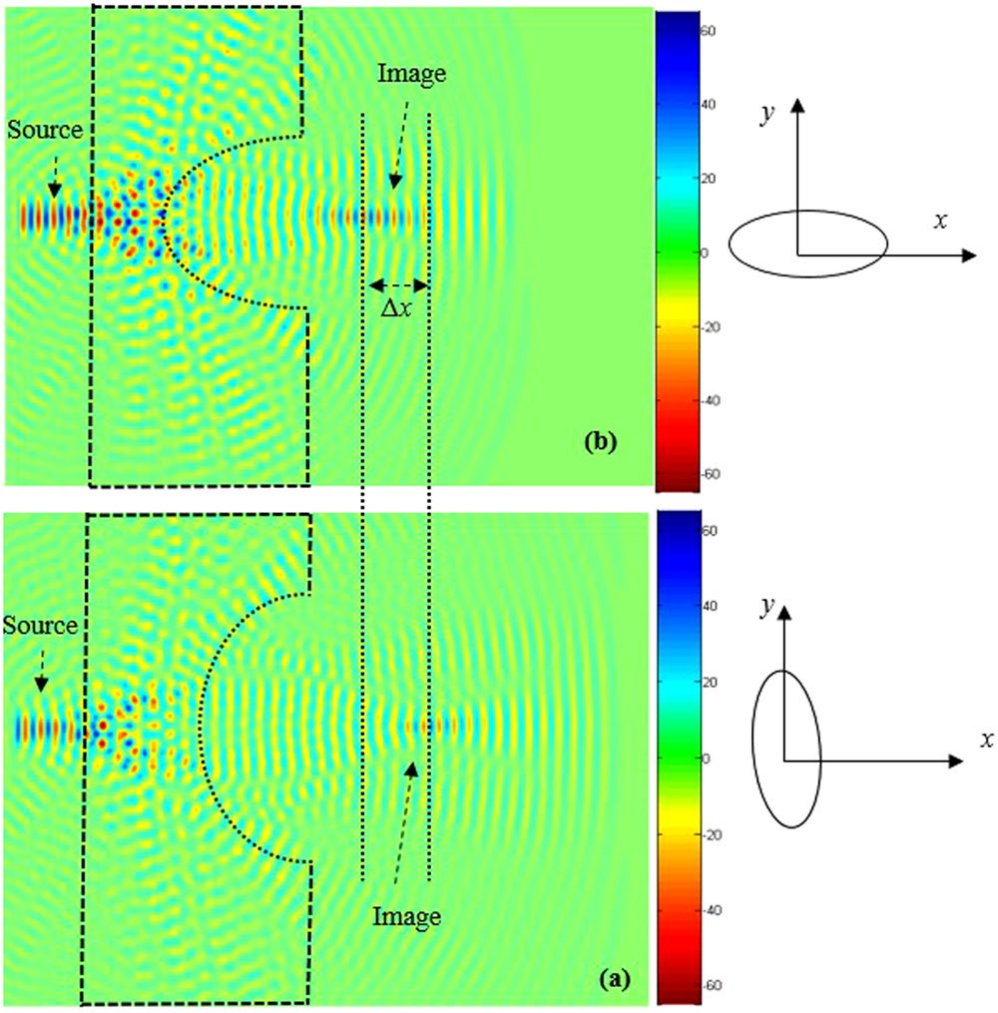
Source and the corresponding image formed by the superlens for a source at wavelength *λ*_0_ = 0.483 μm when the concave region of the superlens is elliptical: (**a)** When the major axis of the elliptical defect is along the *x*-axis, (**b)** When the major axis of the elliptical defect is along the *y*-axis.

To improve image quality (minimize losses), we introduce reflectors at the edges of the concave lens (shown in Fig. 4). Since in the absence of reflectors, the quality of the image is not clear, as illustrated in Fig. 6. The introduced reflectors contribute to reduce the losses of the light propagation, therefore the beam converges to the plane image which enhances the image quality. It should be stated that the curvature of the lens affects the number of the defects, and therefore influences the quality of the formed image. The introduction of reflectors converge the light to the image centre.

It is clear that enhancement of image resolution is possible if the superlens is concave, and it may be possible to use it as a magnifying superlens in terms of resolution and image location. Based on these properties, we investigate the electromagnetic response of the PC structure when the concave region of the superlens is elliptical. We study the effect of the radius of the ellipse and its orientation on the displacement of the image, while keeping the fixed source at the distance *a* = 142 nm. The following structure geometrical parameters are consider; the length of the minor axis of the ellipse along *x*-direction is 4*a* and the length of the major axis along the *y*- direction is 5*a*. The electric field snapshot is illustrated in Fig. 6. We can clearly notice that a sharp image of the object (the source) is formed. By reversing the orientation of the ellipse: i.e the major and the minor axis are along *x* and *y* direction, respectively, the image is shifted along *x*- direction with a distance Δ*x* = 142 nm, as shown in Fig. 6b). Based on the dispersion relation, effective refractive indices for unit cells with differentrod radii are calculated.

**Figure 7:**
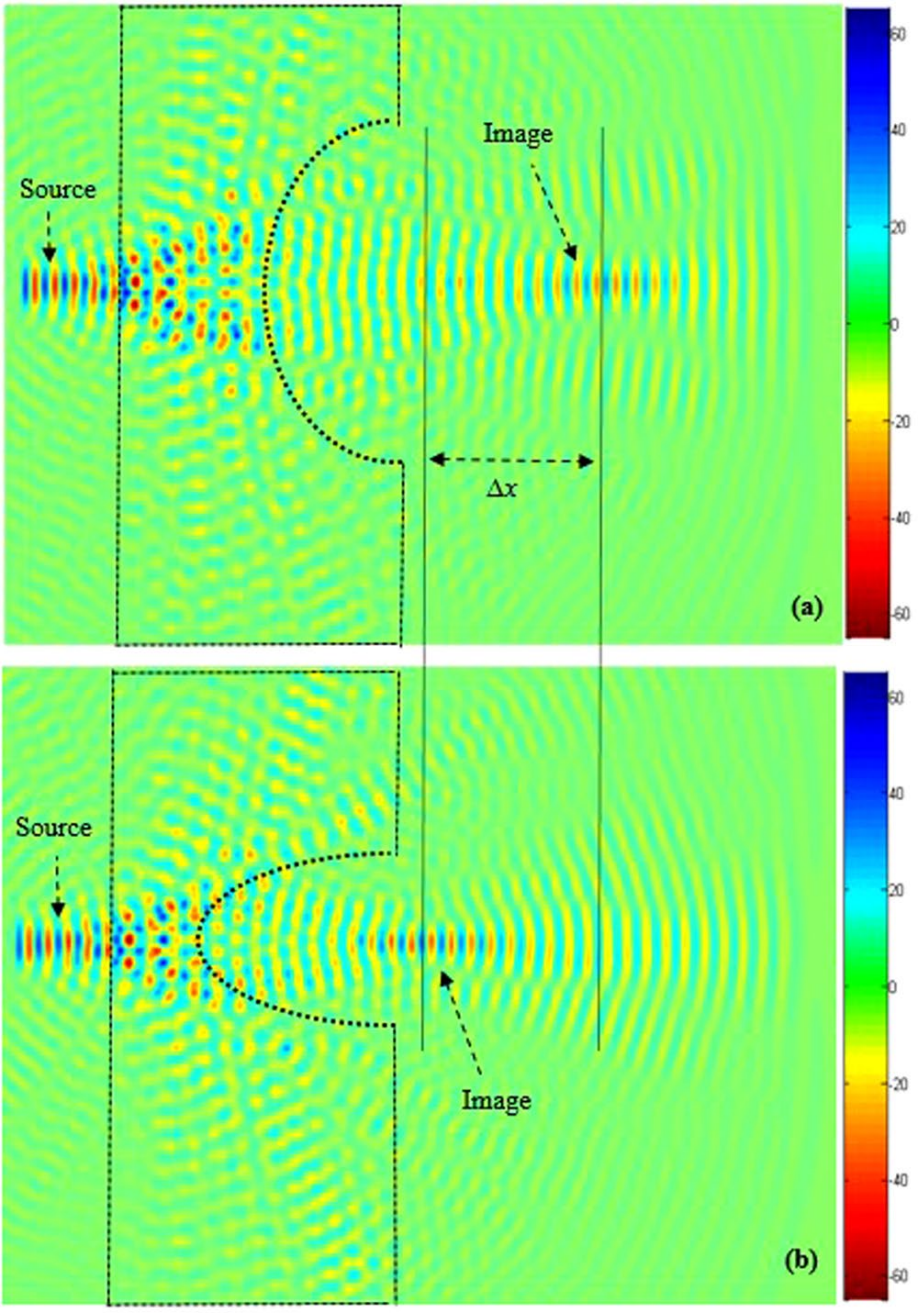
Time domain snapshots of the propagation beam when defects have been introduced in the PC superlens structure in order to realize a perfect imaging. Two different orientation of the elliptical defect are used: (**a)** When the major axis of the elliptical defect is along the *y*-direction, (**b**) When the major axis of the elliptical defect is along the *x-*direction.

In order to shift the image along *x*- direction over long distance (far field), we optimize the dimensions of the ellipse (the minor axis of length 3*a* is following the *x*- direction and the major axis length of 6*a* according to the *y*- direction). Figure 7a shows the position of the image obtained with this configuration. By reversing the orientation of the ellipse, the image is strongly affected and shifts to a longer distance of about Δ*x* = 2*a* = 284 nm. The obtained results are illustrated in the Fig. 7b. When we compare obtained results in Fig. 7 with those in Fig. 6, the translation of the image shows improvements, the image is clearer.

Next, we focus on optimizing the geometric parameters of the ellipse in order to achieve a better lens resolution. We use the following design dimensions: major axis and minor axis lengths of 6*a* and 3*a*, respectively. With this optimal configuration, we obtain a significant offset shift in the image position in the *x*-direction, corresponding to 2*a*.

**Figure 8:**
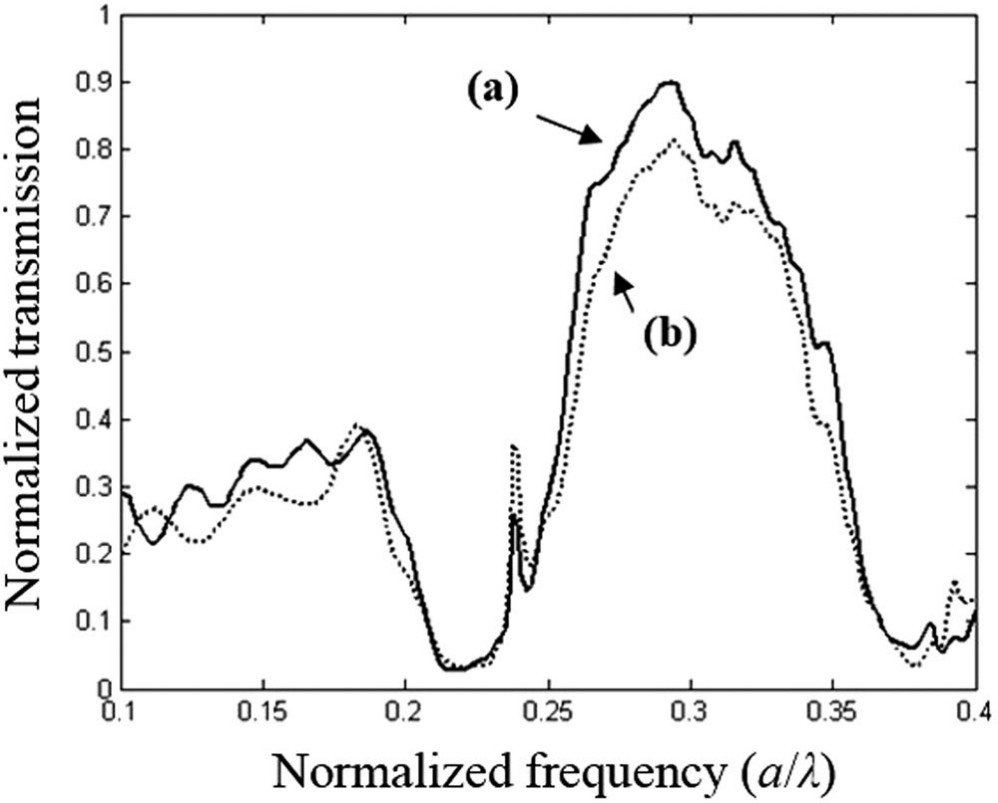
Time-domain simulation of the transmission spectra, when two different orientation of the elliptical defect are used: (**a)** when the major axis of the elliptical defect is along the *y*-direction, (**b)** when the major axis of the elliptical defect is along the *x* direction, the image is strongly affected and shifted to a longer distance of about Δ*x* = 284 nm in the *x*-direction.

In Fig. 8 transmission spectra (calculated by deploying the FDTD method) is shown for the PC superlens structure geometries illustrated in Fig. 7a,b.

**Figure 9:**
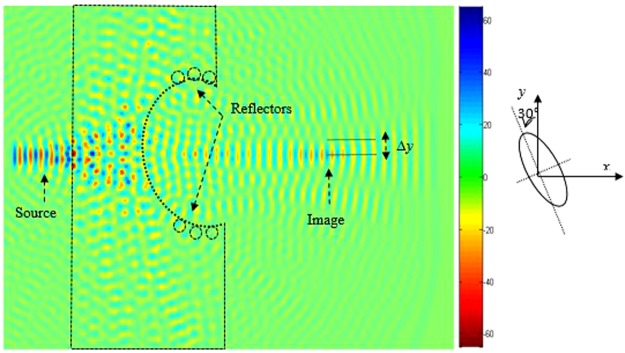
Time-domain simulation showing the imaging effect for a rotation angle *θ* = 30° of the elliptic defect in the anticlockwise direction. The image is shift down along *y*-direction with a distance −142 nm, and is clearly shown in its location.

**Figure 10:**
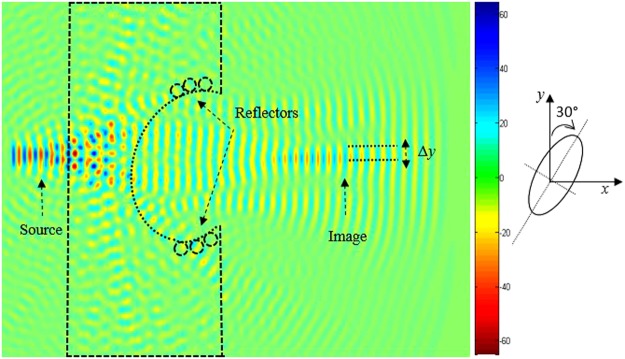
Time-domain simulation showing the imaging effect for a rotation angle *θ* = 30° of the elliptic defect in the clockwise direction. The image is shift upwards along *y*-direction with a distance of 142 nm. A schematic of the elliptical defect orientation is also indicated.

Next, we further investigate and optimize the geometry of the ellipse in terms further improving the image resolutions. Figure 9 illustrates the image positions for two orientations of the ellipse as follows: The image of the position when the major axis is along *y*-direction and the small axis is along *x*-direction, whereas Fig. 10 illustrates the reversed case.

**Figure 11:**
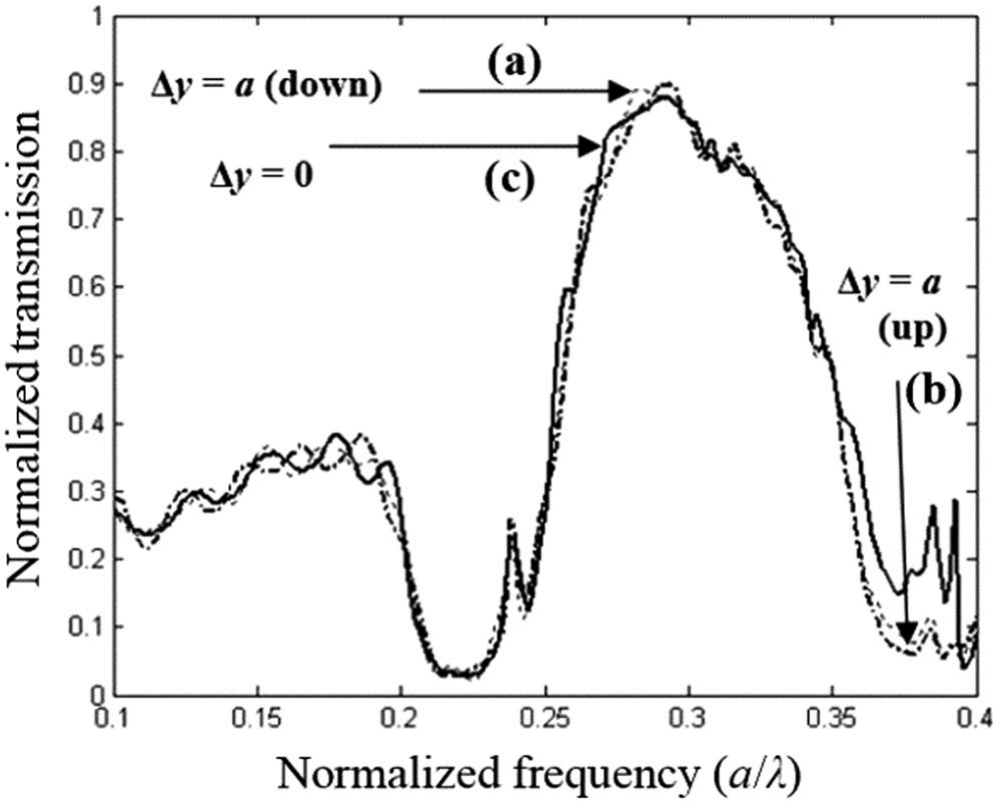
Time-domain simulation of the transmission spectrum, when two different orientation of the elliptical defect are used: (**a)** when the orientation angle of the elliptical defect is *θ* = 30° on the right, the image is strongly affected and shifted down to a longer distance of about Δ*y* = −142 nm along the *y*-direction, (**b**) when the orientation angle of the elliptical defect is *θ* = 30° on the left, the image is strongly affected and shifted up to a longer distance of about Δ*y* = 142 nm along the *y* direction, (**c)** when the major axis of the elliptical defect is along the *y*-direction (*θ* = 0°) which corresponds to the initial position of the image.

To further check the defect shape behaviour of the structure shown in Fig. 7, we calculate the transmission spectra and the obtained results are illustrated in Fig. 11. As can be seen from this figure, the normalised transmission peak of 90% is achieved at the normalised frequency 0.29 *a/λ* when the long axis of the elliptical defect is along the *y*-direction. However, for configuration when the long axis is along the *x*-direction, a slightly lower transmission of 80% is achieved at the same normalised frequency of 0.29 *a/λ*.

To study the elliptical defect parameters and their influence on the vertical image shift, we slightly modify the orientation of the defect, whereas the shape is maintained the same. We have considered a set of numerical simulations of the light propagation for two values of the angle between the long axe of the elliptical defect and the *y*-axes as depicted in Figs 9 and 10. It is obvious from this that when the angle *θ* is equal to 30° in the anticlockwise direction, the image is translated in vertical direction by a distance equal to 142 nm (the image is shift down). However, the image is shifted with the same value (142 nm) and for the same value of angle *θ* but in the clockwise direction, the image is shift upwards, as shown Fig. 10.

In Fig. 11. We illustrate the FDTD calculation of the normalised transmission response of the PC structure for the both value of the rotation angle *θ* that achieve a strong convergence of the lens, operating at the visible frequencies. As shown in this figure, there is a shift of the image upwards with a distance of *Δy* = 142 nm (up) when the rotation angle *θ* is equal to 30°.

A shift in the downwards with a distance Δ*y* = −142 nm (dawn) is obtained when angle *θ* is equal to 30° in the indirect sense. The spectra for Δ*y* = 0, is calculated when the angle *θ* is equal to zero. Moreover, if we compare these spectra, we note that the amplitude of the light is significantly higher from 0, 25*a/λ* to 0.35 *a/λ*, which shows that the field is coupling efficiently in the PC structure with defect due to negative refraction.

**Figure 12:**
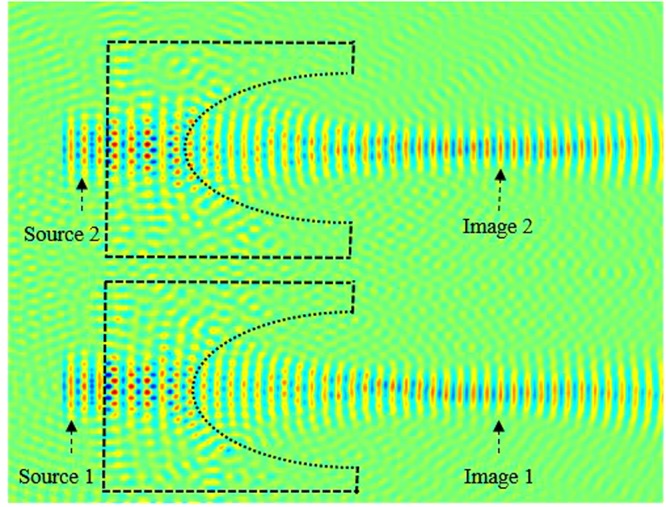
Snapshot of the electric field when the thickness (*t*) of the source is set to *t* = 1.6*a*.

**Figure 13:**
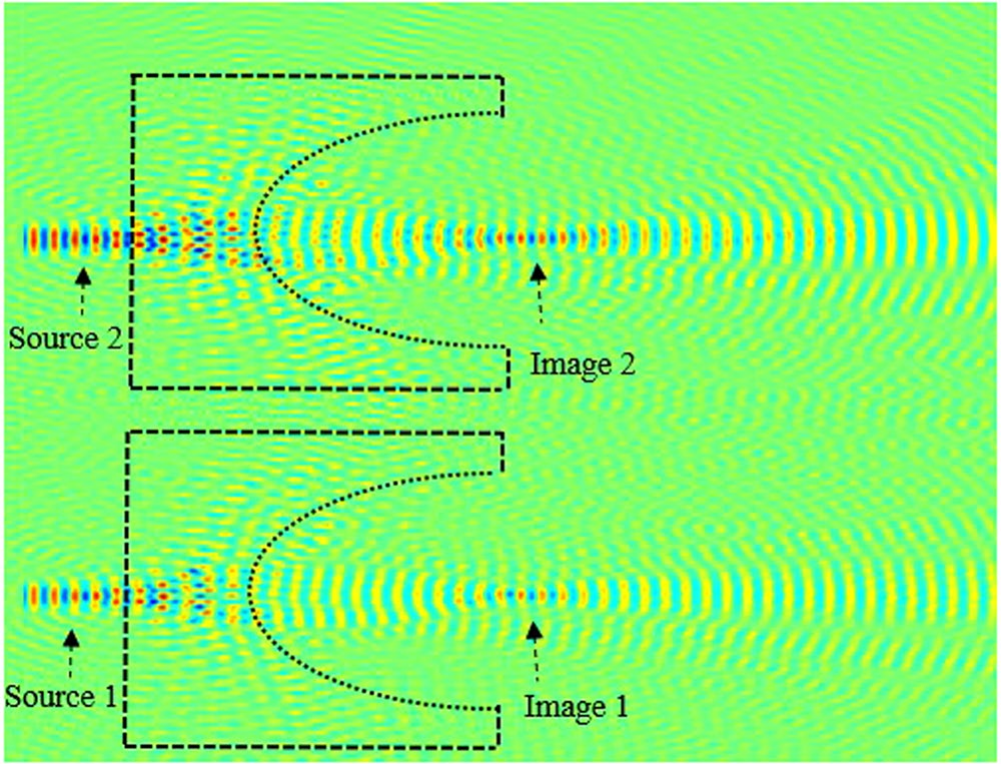
Snapshot of the electric field when the thickness (*t*) of the source is set to *t* = 3.6*a*.

Next, we study the expansion of the source spot size on the images overlapping, in Fig. 12 we illustrate the snapshot of the electric field when the thickness (*t*) of the source is set to 1.6*a*. It can be clearly seen that the formed images are confined into the plane and not coupled to each other. In case when we increase *t* = 3.6*a*, the snapshot of the images are less confined with intensities decreases dramatically compared to the source as shown in Fig. 13. The reason for the decreases of the image intensity is that the excited energy expands over wide range of photon energy resulting in less confinement which affects the image. It can be noticed that, the focal length is 2504.6 nm away from the concave face, which is smaller than the focal length (around 25 μm) reported in ref.^41^.

**Figure 14:**
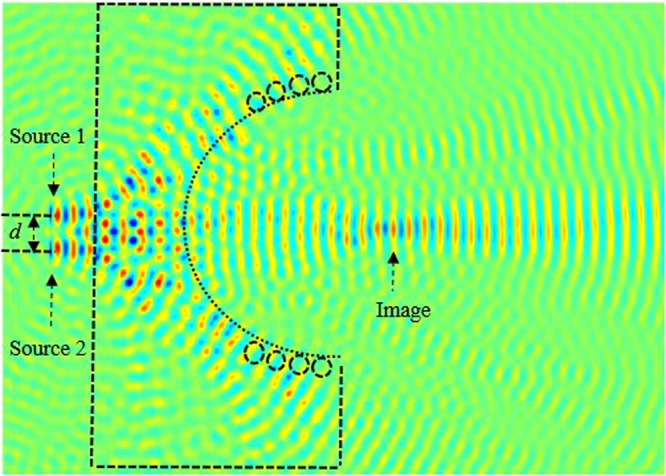
Schematics of the image when sources are separated by distance *d*.

**Figure 15:**
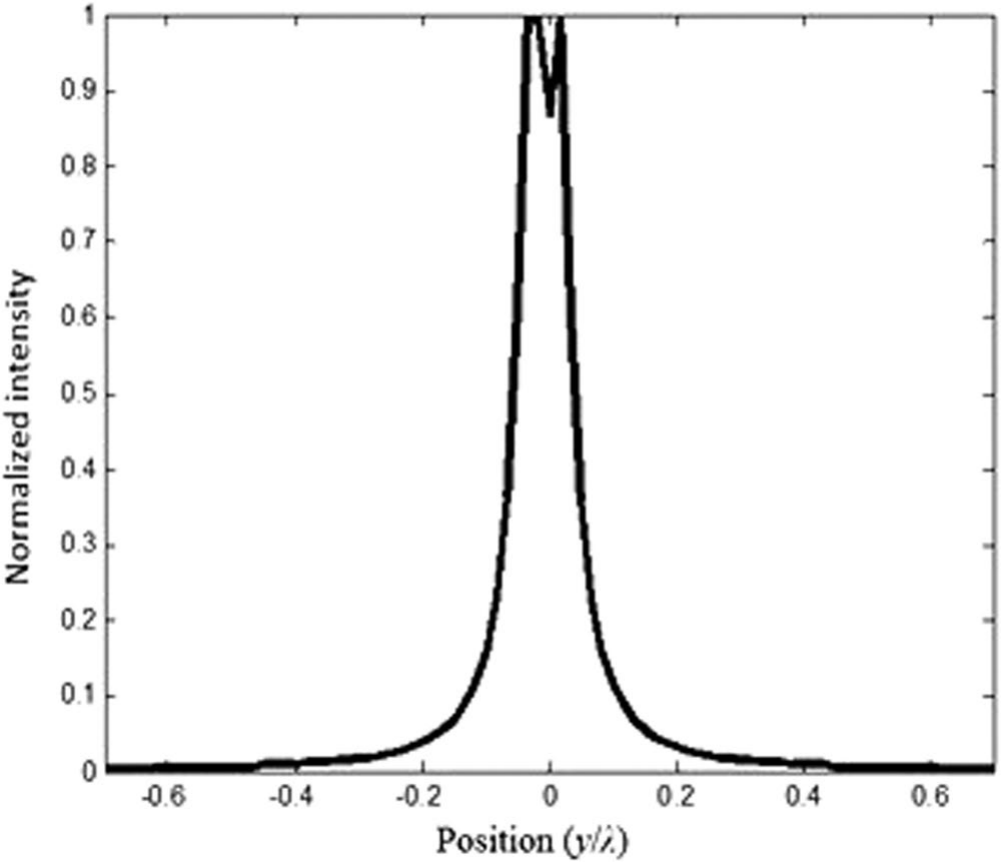
Illustration of the image interference when the separation distance *d* = 0.

Next, in order to analyze and obtain higher quality resolution, we kept *t* = 1.6*a*, and we placed a second source (source 2) parallel to the source 1, as illustrated in Fig. 14. Source 1 and source 2 are separated by a distance *d*. We investigate the image intensity image interference at the focal length by optimizing the separation distance between two sources. In in Fig. 15, we have illustrated the light intensity when the separation distance between two sources is *d* = 0. It is clear from our calculations shown in Fig. 15, that the images are not separated, they are overlying on each other.

**Figure 16:**
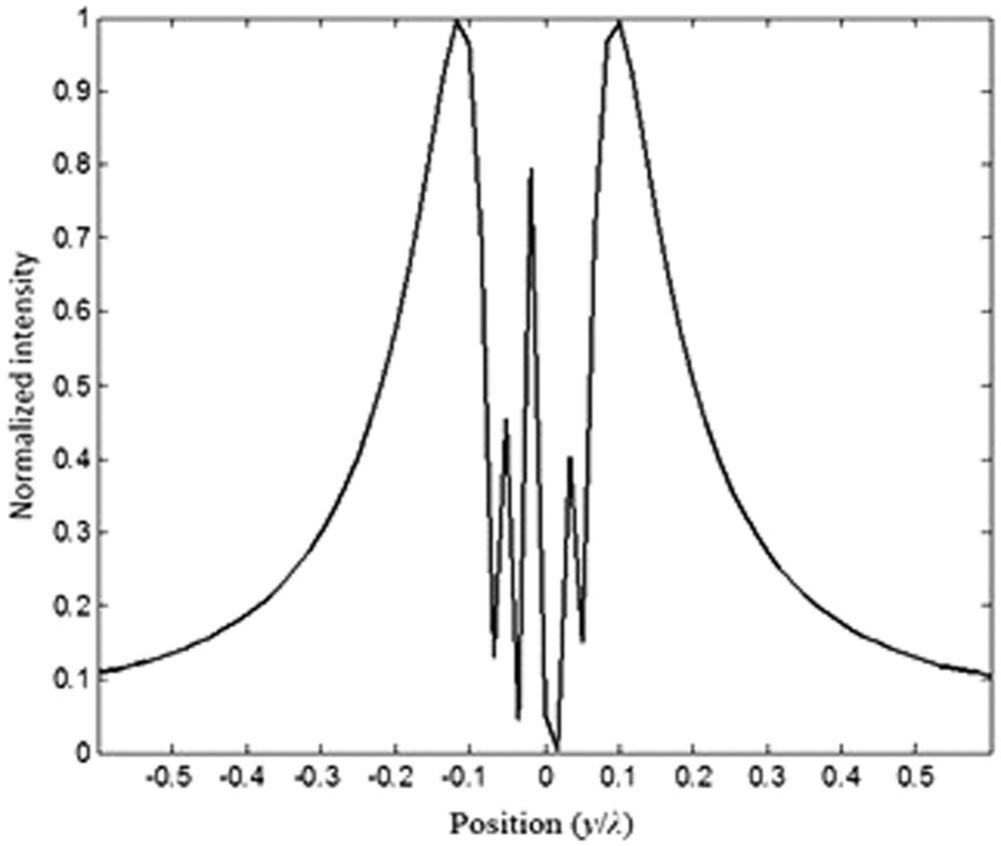
Illustration of the image interference when *d* = 0.55*λ*.

Next, we further investigate and optimize the effect of distance *d* on the image interferences. We increase the distance between sources *d* to 0.55*λ*, as illustrated in Fig. 16. From this figure, it can be clearly seen that the image intensities are still interfering with each other. This shows that the sources are not separated enough, their spot sizes still interact preventing the separation.

**Figure 17:**
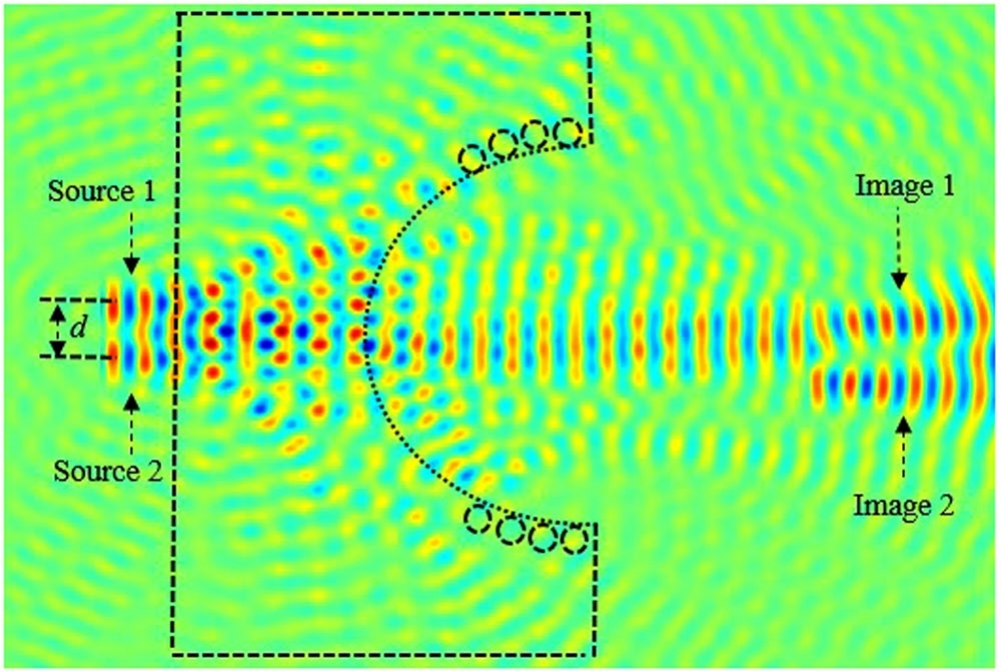
Snapshot of the electric field when the separation distance *d* is set to *d* = 0.56*λ* and *t* = 1.6*a*.

**Figure 18:**
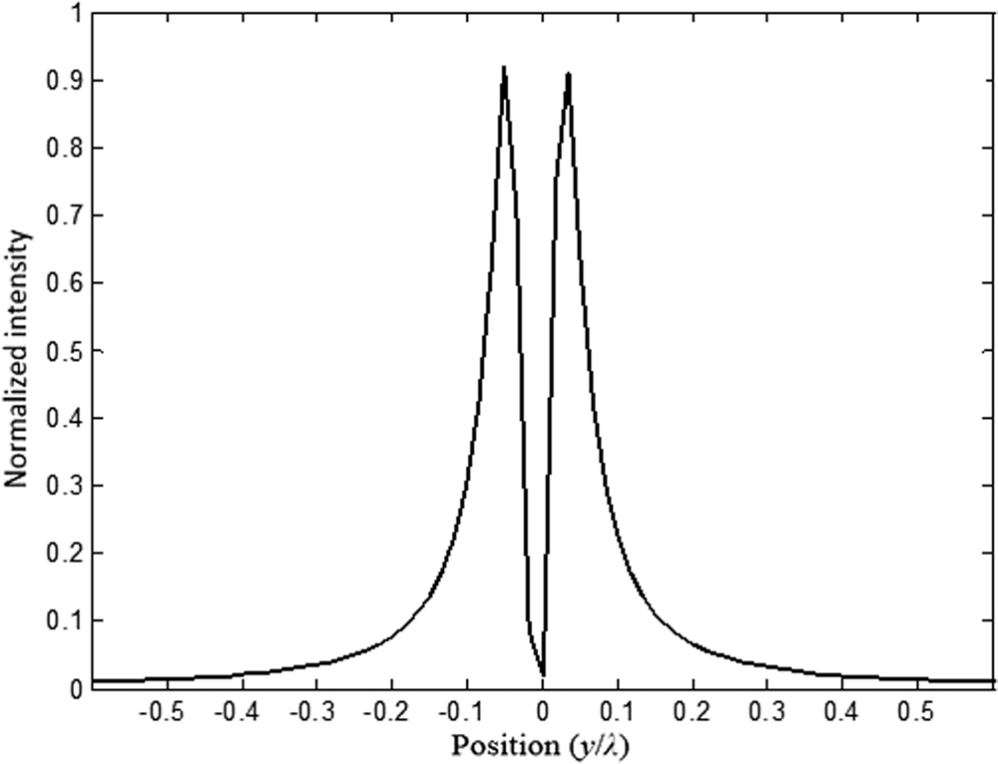
Illustration of the image interference when *d* = 0.56*λ*.

We keep increasing (optimizing) *d* which is set to 0.56 *λ*. The image intensities along with the electrical field image snapshots are calculated and illustrated in Figs 17 and 18, respectively.

The FWHM comparison for the above structure geometries (illustrated in Figs 3, 4, 6a,b, 7a,b, 9, 10 and 14 are illustrated in Table 1. The highest FWHM is Fig. 2, due the fact that reflectors are not yet introduced, and also due to the existence of the two light sources. One can see that FWHM changes slightly in Figs 4, 6a,b, 7a,b, 9 and 10, these values are close to each other due to the angle *ϴ* which displaces the image along *x* and *y* directions, therefore there is not so much effect on the light intensity.

**Table 1:** Obtained FWHM for different structure geometries.

Figure	3	4	6a	6b	7a	7b	9	10	14
FWHM (nm)	1120	520	526	531	529	533	514	519	601

From our calculations of the intensity at the plane image as a function of the position, illustrated in Fig. 18, it is clear that the formed images are separated and not coupled anymore, therefore the desired resolution is realized by optimizing the thickness (*t*) and the distance *d* between the two sources. This can also be confirmed from the snapshots of the electric field shown in Fig. 16. It is clear from both Figs 17 and 18, that the images are no longer coupled.

**Figure 19:**
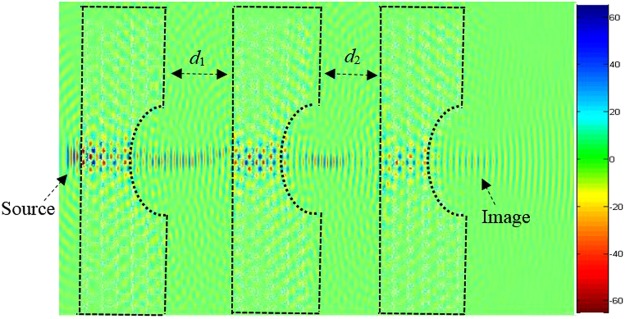
Snapshot of the electric field for three lenses when *d*_1_ = *d*_2_ = 188 nm.

In order to investigate the length of the arrayed structure, we consider three sources separated by *d*_1_ and *d*_2_. Initially, we kept fixed the following lens array design parameters; *d*_1_ = *d*_2_ = *f* = 1500 nm = 3.1 λ, and performed the evolution of the electric field along the array. The snapshot of the electric field evolution is illustrated in Fig. 19. It is evident form this figure, that the image is as clear as the source. Next, we have decreased the distance *d*_1_ = *d*_2_ = *f = *750 nm = 3.1 *λ*/2, the obtained image is has been affected, the image quality has been reduced. Subsequently, we further decreased the lens array parameters; *d*_1_ = *d*_2_ = *f* = 375 nm = 3.1 *λ*/4, and *d*_1_ = *d*_2_ = *f* = 188 nm. It is evident from our calculations that the image resolution quality is very low, in other words the quality of the image is reduced significantly when compared to obtained image when *d*_1_ = *d*_2_ = *f* = 1500 nm = 3.1 λ. Therefore based on our simulations, in order to obtain a high quality image resolution, the light sources should be kept at equidistance with the following parameters; *d*_1_ = *d*_2_ = *f* = 1500 nm. In order to further analyse the image quality we have performed field intensity simulations for various distances between lenses, such as *d*_1_ and *d*_2_. Our study shows that when *d*_1_ and *d*_2_ are equal to 1500 nm its maximum is at about 16 a.u, the width of the field intensity is narrow, also the electric field propagates via longer distance where the losses are small, and therefore the image quality is higher. However, when the distances *d*_1_ and *d*_2_ decrease, the intensity decreases and its width becomes more broaden and the formed image is affected as shown in Fig. 19 when *d*_1_ = *d*_2_ = 188 nm.

**Figure 20:**
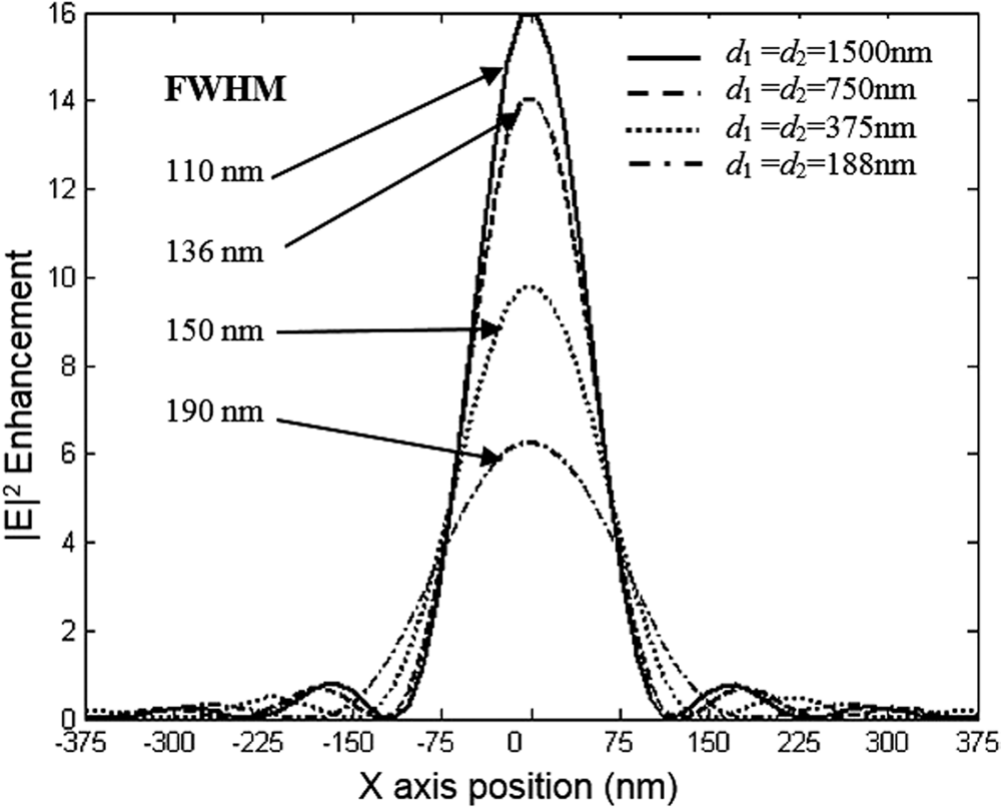
FWHM for various values of lens distances *d*_1_ and *d*_2_.

We have also calculated the light intensity E^2^ for different values of *d*_1_ and *d*_2_ where we determine the FWHM, as shown in Fig. 20. As can be seen from this figure, the light intensity E^2^ has been enhanced significantly when *d*_1_ = *d*_2_ = 1500 nm.

Based on the above proposed design, many other superlenses with desired image projection and resolution can be designed and integrated into a single platform^27^. Integration of the proposed superlenses into a single platform with precise accuracy will have significant effect on creating new M-Lens that will be consisted of a number of such superlens where each superlens could have different design constructions with precise required/desired image projection. In this regard, the proposed superlens has significant advantages when capturing the pixels size of and object. The proposed superlens can be designed even as small as the pixel size. Current conventional microlens arrays used for imaging suffer from high light losses, low (poor) resolution, cannot determine the depth in third dimensions, they have limited angle of view. It is also known that the resolution of current optical devices is limited by the light operating wavelength. In particular, any current conventional lens cannot be fabricated and are unable to deal with images of the objects smaller than the operating wavelength. So, the sharpness of the image of the conventional lens is limited by the light operating wavelength. In order to achieve a strong coverage of the beam, several lenses should be integrated on the single chip, therefore the size of the device would be increased. Also when several lenses are integrated in a single platform the design of each lens should be optimized and adjusted in order to maintain the image resolution quality.

## Conclusion

In this paper, we have demonstrated an ultra-compact imaging lens based metamaterial PC structure operating at visible wavelength. The proposed structure consists of a GaP/Ag/GaP metamaterial with a concave geometry shape with defects. We have used 2D FDTD numerical simulations to design and investigate a feasibility study of the proposed superlenses and integrate some of them in a single platform at operating visible wavelength. A comprehensive analysis of the geometric superlens and their integration parameters are performed. It is found that the lensing shift of the image in both directions, for each superlens, is strongly dependent on the angle and the shape of the introduced defect. The influence of the angle and shape of defects and the distance between integrated superlenses on the quality of the image resolution have been analyzed. Effects of the light coupling between integrated lenses on image quality resolution has been analyzed. Our initial feasibility model clearly demonstrates the proof-of-concept (POC) which verifies that the proposed idea have the potential to revolutionize the imaging technology for real-world light field imaging application. In this POC, we have demonstrated visible light control in 2D plane (*x* and *y*) imaging. Controlling and manipulation of the light in desired 3^rd^ dimension for each integrated lens, while maintaining high imaging resolution, is challenging, therefore our future study will be focused on 3D design and integration of the proposed superlenses, in a single platform, with high imaging resolution, operating at visible wavelength.
